# Mast Cells: Fascinating but Still Elusive after 140 Years from Their Discovery

**DOI:** 10.3390/ijms21020464

**Published:** 2020-01-11

**Authors:** Gilda Varricchi, Gianni Marone

**Affiliations:** 1 Department of Translational Medical Sciences (DISMET), University of Naples Federico II, 80100 Naples, Italy; 2 Center for Basic and Clinical Immunology Research (CISI), University of Naples Federico II, School of Medicine, 80100 Naples, Italy; 3 WAO Center of Excellence, 80100 Naples, Italy; 4 Institute of Experimental Endocrinology and Oncology “Gaetano Salvatore” (IEOS), National Research Council (CNR), 80100 Naples, Italy

**Keywords:** angiogenesis, atherosclerosis, cancer, cardiovascular diseases, cysteinyl leukotrienes, lymphangiogenesis, macrophages, mast cells, mastocytosis

## 1. Preface

**Figure 1:**
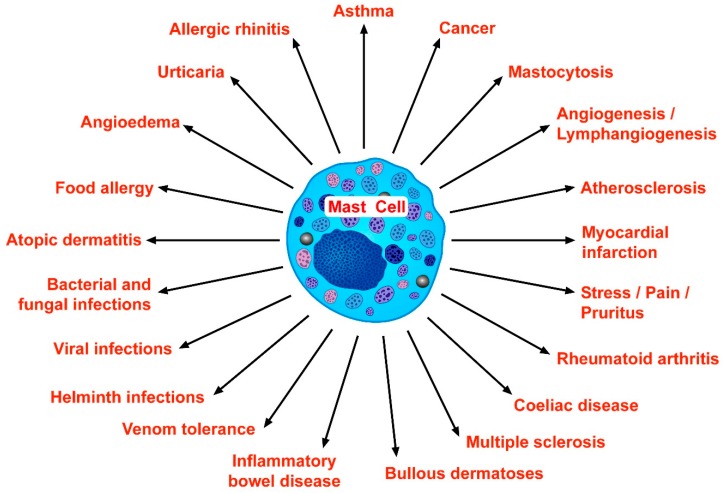
This figure schematically illustrates the wide spectrum of pathophysiological conditions in which mast cells and their mediators have been implicated. For several decades mast cells were considered to play mainly proinflammatory roles in several allergic disorders, such as bronchial asthma [2,3,4,122], allergic rhinitis [5], urticaria [6,7], food allergy [8,9], anaphylaxis [10,11], atopic dermatitis [12], and angioedema [13]. During the last years, it became evident that mast cells represent an important cell during bacterial [26,27,29], fungal [29], viral [25,29], and helminth infections [30,31]. Elegant studies have demonstrated that mast cell-derived mediators can play protective roles against several venoms [114,115]. Mast cells and their mediators can be involved in several aspects of tumor initiation and growth [21,39,69,70,71], presumably through the production of several angiogenic and lymphangiogenic factors [19,20,75]. Systemic mastocytosis is a clonal disease associated with a somatic gain-of-function *KIT* mutation [56,57,123]. Mast cells, strategically located in different sections of the human heart [51,52] and atherosclerotic plaque [32,33], are involved in different phases of atherosclerosis and myocardial infarction. These cells can be involved in several autoimmune disorders, such as rheumatoid arthritis [37], coeliac disease [94], multiple sclerosis [124], and bullous dermatoses [125]. Mast cell–nerve communications are involved in stress, pain, pruritus [126,127], and in inflammatory bowel diseases [35,36].

Some of the basic characteristics of tissue mast cells were described over 140 years ago by Paul Ehrlich, the founder of modern immunology [1]. At that time, the mast cells’ distinguishing feature was the affinity of their cytoplasmic granules for certain basic dyes. For several decades, mast cells and their mediators were essentially considered to play mainly a proinflammatory role in allergic disorders, such as bronchial asthma [2,3,4], allergic rhinitis [5], urticaria [6,7], food allergy [8,9], anaphylaxis [10,11], atopic dermatitis [12], and angioedema [13]. With the appreciation of these cells as major potential sources of a myriad of cytokines and chemokines, it became evident in the 1990s that mast cells may express immunoregulatory functions [14,15]. During the last decades, it was demonstrated that mast cells can also produce different proangiogenic [16,17,18] and lymphangiogenic factors [19,20], suggesting that they may actually play a role in tumor initiation and growth [21,22,23,24]. Moreover, these cells can be activated by different viral [25,26] and bacterial proteins [27,28] and thereby represent a potentially important cell during microbial infections. Therefore, the spectrum of diseases in which mast cells and their mediators have been implicated has extended to include bacterial, fungal, viral, and helminth infections [26,29,30,31]; several diseases of the cardiovascular [14,32,33] and gastrointestinal systems [34,35,36]; and the joints [37,38]. Figure 1 schematically summarizes the wide spectrum of pathophysiological conditions in which mast cells and their mediators have been implicated during the last decades.

This volume contains contributions by several established investigators in the field of mast cell biology. The volume starts with a collaborative paper by Stephen J. Galli, Gilda Varricchi, and Gianni Marone, illustrating initial and more recent studies which have attempted to identify distinct “subpopulations” of mast cells based on the analyses of transcriptomes of anatomically distinct mouse mast cell populations [39,40,41,42]. The authors illustrate the important roles played by mast cells to the control of homeostasis in different pathophysiological conditions. Moreover, they discuss the possibility that distinct subpopulations of mast cells could play different roles in cardiovascular disorders and in tumorigenesis. Finally, the authors speculate that at least two major subsets of mast cells, MC1 and MC2, like macrophages (M1 and M2 subtypes) [43], dendritic cells (D1 and D2) [44], and neutrophils (N1 and N2) [45,46], could play distinct or even opposite roles in different pathophysiological conditions.

Kirshenbaum and collaborators describe the biochemical and immunological characteristics of a novel human mast cell line (LADR) which they have established [47]. LADR cells are characterized by a slower proliferation rate and more advanced development compared to the classical LAD cell line. This new cell line appears to be a valuable addition for in vitro studies of human mast cell biology.

Mekori and coworkers illustrate the possible roles of various miRNAs in IgE-mediated allergic and non-allergic diseases involving mast cell activation [48]. Theoharides and collaborators report that IL-27, produced by activated macrophages, can be modulated by mast cell mediators, such as heparin and tryptase [49]. Kwon and Kim report that leukotriene B_4_ (LTB_4_) can activate the low-affinity LTB_4_ receptor, BLT_2_, on mast cells. Engagement of BLT_2_ mediates the synthesis of the most potent proangiogenic molecule, vascular endothelial growth factor (VEGF-A), and IL-13 from mast cells. The authors speculate that novel strategies aimed to block BTL_2_ could contribute to the treatment of allergic disorders [50].

It is well established that mast cells are strategically localized in different sections of the human heart, such as the myocardium [51,52], the atherosclerotic plaque [33], and the aortic valve [53]. Kovanen comprehensively reviews the complex role of mast cells throughout the progression of early to late lesions of human atherosclerosis [32]. Immunohistochemical studies in autopsied patients and studies in cell culture systems and in atherosclerotic mouse models have collectively provided evidence that mast cell mediators may promote atherogenesis at various stages of lesion development.

Mastocytosis is a hematopoietic neoplasm characterized by abnormal expansion and focal accumulation of clonal mast cells in various organs [54,55,56]. The disease is highly heterogeneous and exhibits a complex pathology and different clinical presentations. Valent and a group of international leaders reviewed the WHO classification of mastocytosis and their different prognosis. The authors also illustrate the different symptoms and associated co-morbidities of various forms of mastocytosis. Finally, they emphasize the multidisciplinary aspects of the disease and discuss related challenges in daily practice [57]. Another group of mastocytosis experts demonstrate the expression of programmed death ligand 1 (PD-L1) on mast cells from patients with mastocytosis [58]. PD-L1 is expressed on tumor cells [59,60] and also on several activated immune cells, including CD4^+^ and CD8^+^ T cells, B cells, NKT cells, and mast cells [61,62,63]. PD-L1 expression has been shown to be upregulated in several tumor cells as a mechanism of immune suppression and evasion [64]. The authors review the literature on PD-L1 expression on mast cells from patients with mastocytosis.

Aldehyde dehydrogenase 2 (Aldh2) is the most efficient isoenzyme within the ALDH enzymes to remove toxic metabolites from the metabolism of alcohol [65]. A genetic polymorphism (rs671) in ALDH2 is present in approximately 40% of Eastern Asian populations [65,66] and is associated with alcohol flush syndrome [67]. Kim and coworkers demonstrate that bone-marrow-derived mast cells from mice with a genetic deletion of *Aldh2* have increased proliferation and IL-6 production after activation by stem cell factor (SCF), as well as when co-stimulated with SCF and an antigen [68]. These findings provide insight into the regulation of mast cell responsiveness in relation to alcohol-associated flushing.

There is increasing evidence that mast cells and their mediators can be involved in several aspects of tumor initiation and growth [21,39,69,70]. However, their impact on experimental and human tumors remains controversial [22,23]. Several papers in this volume address this complex and still controversial issue. Redegeld and collaborators, by using a 3D co-culture model, elegantly investigated the role of mast cells in colon cancer. By comparing the transcriptomic profile of colon cancer-co-cultured mast cells versus control mast cells, they identify several deregulated genes which can contribute to cancer development. This experimental model could represent a novel approach to investigate the role of mast cells in tumorigenesis [71].

Sammarco and collaborators investigated the role played by mast cells in the modulation of angiogenesis and lymphangiogenesis in human gastric cancer [21]. They report that mast cell density is increased in gastric cancer and there is a correlation with angiogenesis [72,73]. They also report that gastric mast cells express PD-L1, a relevant checkpoint, and that several undergoing clinical trials are targeting immune checkpoints in gastric cancer. The authors suggest that elucidation of the role of subsets of mast cells in different human gastric cancers will demand studies of increasing complexity beyond those assessing merely mast cell density and microlocalization. Antonelli and coworkers, based on their long-lasting experience, comprehensively reviewed the roles of immune and inflammatory cells, cytokines, and chemokines in the thyroid cancer microenvironment [74]. Ribatti and Vacca illustrate the role of bone marrow angiogenesis in the pathogenesis and progression of hematological malignancies [75]. Based on their extensive experience, they discuss the roles played by mast cells in the modulation of angiogenesis in patients with multiple myeloma. Sagi-Eisenberg describes a novel mechanism by which adenosine, released by activated mast cells, can autocrinally activate the A3 adenosine receptor [76].

Mast cells are strategically located at sites that interface with the external environment, such as the skin [77], lung [78], and intestine [34,79]. These locations allow mast cells to act as sentinels for tissue damage and pathogen invasion [4]. Moreover, the association between mast cells and blood vessels [32,52] is optimal to foster the rapid recruitment of immune cells out of the bloodstream and into the inflamed tissues. This process is facilitated by the mast cell production of TNF-α [80,81,82,83,84] and IL-1β [85,86] that activate endothelial cells, the release of vasoactive mediators (i.e., histamine and cysteinyl leukotrienes) [87,88], and chemokines that promote the recruitment of inflammatory and immune cells [24,70,89,90,91,92]. Marshall and coworkers elegantly reviewed the complex roles of mast cell responses to viruses and pathogen products [26]. This review highlights the complexity of mast cell biology in the context of innate immune responses. Di Nardo and collaborators elegantly demonstrated that mast cells express lipocalin 2 (LPCN2), a known inhibitor of bacterial growth. Using mast cells derived from mice deficient in LPCN2, they show that this antimicrobial peptide is an important component of mast cell activity against *Escherichia coli.* They also demonstrate that sphingosine-1-phosphate (S1P) activates a specific receptor (S1PR) on mast cells to release LPC2, which exerts antimicrobial activity against several bacteria such as *Staphylococcus aureus* and *E. coli* [93]. Piliponsky and collaborators extensively reviewed the role of mast cells and their mediators in viral, bacterial, and fungal infections [29]. They discuss recent studies focused on mast cell interactions with flaviviruses and *Candida albicans,* and mast cell functions in a model of cecal ligation and puncture. Collectively, the results of these studies illustrate that mast cells can either promote host resistance to infections or contribute to a dysregulated host response that can increase host morbidity and mortality.

Coeliac disease is a human autoimmune-like disorder characterized by chronic inflammation of the small intestine induced by proline- and glutamine-rich wheat gluten [94,95]. Coeliac disease is the result of complex interactions of genetic, environmental, and immunological factors [96]. Although coeliac disease is considered a prototype of T-cell mediated disease [96], the innate immune system can contribute to its pathogenesis. Frossi and collaborators’ review has interesting results, indicating that mast cells and their mediators could play a role in the pathogenesis of coeliac disease [94].

Rheumatoid arthritis is a chronic systemic autoimmune disease primarily affecting the joints [97]. Mast cells are present in healthy synovial tissue [98] and their density is increased in rheumatoid arthritis synovitis [99,100]. However, the exact functions and the correlations of mast cell density with disease development and progression are still largely unknown. Moreover, contradictory data have been obtained in animal models and from patients with long-lasting disease [101,102,103]. Rivellese and coworkers present a careful revision of the literature on mast cells in rheumatoid arthritis, including recent observations from patients with early disease indicating that these cells are relevant markers of disease severity [37,38].

In recent years, accumulating evidence has revealed the close anatomical contact and functional interactions between neurons and mast cells [104,105,106]. Theoharides and coworkers present a careful revision of the literature and recent findings on mediators released from activated mast cells that could activate microglia [107,108], causing localized inflammation [109,110,111] and some symptoms of autism spectrum disorder [112].

Boo and collaborators present original results in a mouse model of allergen-provoked localized vulvodynia, supporting the hypothesis that mast cells are involved in this painful disorder [113].

## 2. Conclusions and Future Directions

This is a wonderful time in mast cell research. Indeed, the last years have witnessed unprecedented progress in our understanding of the development of mast cells [40,41,42]. Moreover, extraordinary progress has been made in understanding the complex homeostatic and protective roles of these cells in different pathophysiological conditions [31,39,114,115]. Mast cells, known for decades for their detrimental role in allergic diseases, are now recognized to play crucial roles in a diverse array of physiological and pathologic functions [15,30,116]. We would like to speculate that such different, sometime opposite effects of mast cells are made possible by the plurality of mast cell subpopulations. Recently, comprehensive analysis of the transcriptome of individual anatomically distinct mast cells [117] and fate-mapping system [40,41,118] demonstrate that rodent mast cells form a highly heterogeneous population of immune cells [40,41,42], similar to macrophages [43,119] and T cells [120,121]. These fascinating results indicate that much more remains to be discovered in development, migration to tissues, biochemistry, and functions of different subsets of rodent and human mast cells.

After 140 years from their discovery, mast cells remain fascinating but still elusive cells of the immune system. The characterization of subpopulations of mast cells by single-cell RNA-seq, together with analysis of encoded proteins, will be of paramount importance to modulate the injury- or repair-inducing abilities of these immune cells.

## Abbreviations

Aldh2	Aldehyde dehydrogenase 2
BTL_2_	Low-affinity leukotriene (LT) B_4_ receptor
*E. coli*	Escherichia coli
IL-13	Interleukin-13
LPCN2	Lipocalin 2
LTB_4_	Leukotriene B_4_
NKT	Natural killer T-cell
PD-L1	Programmed Death Ligand 1
S1P	Sphingosine-1-phosphate
S1PR	Sphingosine-1-phosphate receptor
SCF	Stem cell factor
TNF-α	Tumor Necrosis Factor-α
VEGF-A	Vascular Endothelial Growth Factor-A

## References

[1] Ehrlich P. (1878). Beitrage sur Theorie und Praxis der Histologischen Farbung. Ph.D. Thesis.

[2] Marone G., Borriello F., Varricchi G., Genovese A., Granata F. (2014). Basophils: Historical reflections and perspectives. Chem. Immunol. Allergy.

[3] Varricchi G., Raap U., Rivellese F., Marone G., Gibbs B.F. (2018). Human mast cells and basophils-How are they similar how are they different?. Immunol. Rev..

[4] Galli S.J., Tsai M. (2012). IgE and mast cells in allergic disease. Nat. Med..

[5] Zhai G.T., Wang H., Li J.X., Cao P.P., Jiang W.X., Song J., Yao Y., Wang Z.C., Wang Z.Z., Wang M.C. (2018). IgD-activated mast cells induce IgE synthesis in B cells in nasal polyps. J. Allergy Clin. Immunol..

[6] Church M.K., Kolkhir P., Metz M., Maurer M. (2018). The role and relevance of mast cells in urticaria. Immunol. Rev..

[7] Siebenhaar F., Redegeld F.A., Bischoff S.C., Gibbs B.F., Maurer M. (2018). Mast Cells as Drivers of Disease and Therapeutic Targets. Trends Immunol..

[8] Macchia D., Melioli G., Pravettoni V., Nucera E., Piantanida M., Caminati M., Campochiaro C., Yacoub M.R., Schiavino D., Paganelli R. (2015). Guidelines for the use and interpretation of diagnostic methods in adult food allergy. Clin. Mol. Allergy.

[9] Noah T.K., Knoop K.A., McDonald K.G., Gustafsson J.K., Waggoner L., Vanoni S., Batie M., Arora K., Naren A.P., Wang Y.H. (2019). IL-13-induced intestinal secretory epithelial cell antigen passages are required for IgE-mediated food-induced anaphylaxis. J. Allergy Clin. Immunol..

[10] Leyva-Castillo J.M., Galand C., Kam C., Burton O., Gurish M., Musser M.A., Goldsmith J.D., Hait E., Nurko S., Brombacher F. (2019). Mechanical Skin Injury Promotes Food Anaphylaxis by Driving Intestinal Mast Cell Expansion. Immunity.

[11] Choi H.W., Suwanpradid J., Kim I.H., Staats H.F., Haniffa M., MacLeod A.S., Abraham S.N. (2018). Perivascular dendritic cells elicit anaphylaxis by relaying allergens to mast cells via microvesicles. Science.

[12] Serhan N., Basso L., Sibilano R., Petitfils C., Meixiong J., Bonnart C., Reber L.L., Marichal T., Starkl P., Cenac N. (2019). House dust mites activate nociceptor-mast cell clusters to drive type 2 skin inflammation. Nat. Immunol..

[13] Oschatz C., Maas C., Lecher B., Jansen T., Bjorkqvist J., Tradler T., Sedlmeier R., Burfeind P., Cichon S., Hammerschmidt S. (2011). Mast cells increase vascular permeability by heparin-initiated bradykinin formation in vivo. Immunity.

[14] Varricchi G., Rossi F.W., Galdiero M.R., Granata F., Criscuolo G., Spadaro G., de Paulis A., Marone G. (2019). Physiological Roles of Mast Cells: Collegium Internationale Allergologicum Update 2019. Int. Arch. Allergy Immunol..

[15] Mukai K., Tsai M., Saito H., Galli S.J. (2018). Mast cells as sources of cytokines, chemokines, and growth factors. Immunol. Rev..

[16] Boesiger J., Tsai M., Maurer M., Yamaguchi M., Brown L.F., Claffey K.P., Dvorak H.F., Galli S.J. (1998). Mast cells can secrete vascular permeability factor/ vascular endothelial cell growth factor and exhibit enhanced release after immunoglobulin E-dependent upregulation of fc epsilon receptor I expression. J. Exp. Med..

[17] Theoharides T.C., Zhang B., Kempuraj D., Tagen M., Vasiadi M., Angelidou A., Alysandratos K.D., Kalogeromitros D., Asadi S., Stavrianeas N. (2010). IL-33 augments substance P-induced VEGF secretion from human mast cells and is increased in psoriatic skin. Proc. Natl. Acad. Sci. USA.

[18] Detoraki A., Staiano R.I., Granata F., Giannattasio G., Prevete N., de Paulis A., Ribatti D., Genovese A., Triggiani M., Marone G. (2009). Vascular endothelial growth factors synthesized by human lung mast cells exert angiogenic effects. J. Allergy Clin. Immunol..

[19] Varricchi G., Loffredo S., Borriello F., Pecoraro A., Rivellese F., Genovese A., Spadaro G., Marone G. (2019). Superantigenic Activation of Human Cardiac Mast Cells. Int. J. Mol. Sci..

[20] Varricchi G., Loffredo S., Galdiero M.R., Marone G., Cristinziano L., Granata F. (2018). Innate effector cells in angiogenesis and lymphangiogenesis. Curr. Opin. Immunol..

[21] Sammarco G., Varricchi G., Ferraro V., Ammendola M., De Fazio M., Altomare D.F., Luposella M., Maltese L., Curro G., Marone G. (2019). Mast Cells, Angiogenesis and Lymphangiogenesis in Human Gastric Cancer. Int. J. Mol. Sci..

[22] Varricchi G., Galdiero M.R., Loffredo S., Marone G., Iannone R., Granata F. (2017). Are Mast Cells MASTers in Cancer?. Front. Immunol..

[23] Theoharides T.C., Conti P. (2004). Mast cells: The Jekyll and Hyde of tumor growth. Trends Immunol..

[24] Melillo R.M., Guarino V., Avilla E., Galdiero M.R., Liotti F., Prevete N., Rossi F.W., Basolo F., Ugolini C., de Paulis A. (2010). Mast cells have a protumorigenic role in human thyroid cancer. Oncogene.

[25] de Paulis A., Florio G., Prevete N., Triggiani M., Fiorentino I., Genovese A., Marone G. (2002). HIV-1 envelope gp41 peptides promote migration of human Fc epsilon RI+ cells and inhibit IL-13 synthesis through interaction with formyl peptide receptors. J. Immunol..

[26] Marshall J.S., Portales-Cervantes L., Leong E. (2019). Mast Cell Responses to Viruses and Pathogen Products. Int. J. Mol. Sci..

[27] Genovese A., Borgia G., Bjorck L., Petraroli A., de Paulis A., Piazza M., Marone G. (2003). Immunoglobulin superantigen protein L induces IL-4 and IL-13 secretion from human Fc epsilon RI+ cells through interaction with the kappa light chains of IgE. J. Immunol..

[28] Genovese A., Bouvet J.P., Florio G., Lamparter-Schummert B., Bjorck L., Marone G. (2000). Bacterial immunoglobulin superantigen proteins A and L activate human heart mast cells by interacting with immunoglobulin E. Infect. Immun..

[29] Piliponsky A.M., Acharya M., Shubin N.J. (2019). Mast Cells in Viral, Bacterial, and Fungal Infection Immunity. Int. J. Mol. Sci..

[30] Shimokawa C., Kanaya T., Hachisuka M., Ishiwata K., Hisaeda H., Kurashima Y., Kiyono H., Yoshimoto T., Kaisho T., Ohno H. (2017). Mast Cells Are Crucial for Induction of Group 2 Innate Lymphoid Cells and Clearance of Helminth Infections. Immunity.

[31] Mukai K., Karasuyama H., Kabashima K., Kubo M., Galli S.J. (2017). Differences in the Importance of Mast Cells, Basophils, IgE, and IgG versus That of CD4(+) T Cells and ILC2 Cells in Primary and Secondary Immunity to Strongyloides venezuelensis. Infect. Immun..

[32] Kovanen P.T. (2019). Mast Cells as Potential Accelerators of Human Atherosclerosis-From Early to Late Lesions. Int. J. Mol. Sci..

[33] Kovanen P.T., Bot I. (2017). Mast cells in atherosclerotic cardiovascular disease—Activators and actions. Eur. J. Pharmacol..

[34] Huber M., Cato A.C.B., Ainooson G.K., Freichel M., Tsvilovskyy V., Jessberger R., Riedlinger E., Sommerhoff C.P., Bischoff S.C. (2019). Regulation of the pleiotropic effects of tissue-resident mast cells. J. Allergy Clin. Immunol..

[35] De Zuani M., Dal Secco C., Frossi B. (2018). Mast cells at the crossroads of microbiota and IBD. Eur. J. Immunol..

[36] Bischoff S.C. (2016). Mast cells in gastrointestinal disorders. Eur. J. Pharmacol..

[37] Rivellese F., Mauro D., Nerviani A., Pagani S., Fossati-Jimack L., Messemaker T., Kurreeman F.A.S., Toes R.E.M., Ramming A., Rauber S. (2018). Mast cells in early rheumatoid arthritis associate with disease severity and support B cell autoantibody production. Ann. Rheum. Dis..

[38] Rivellese F., Rossi F.W., Galdiero M.R., Pitzalis C., de Paulis A. (2019). Mast Cells in Early Rheumatoid Arthritis. Int. J. Mol. Sci..

[39] Varricchi G., de Paulis A., Marone G., Galli S.J. (2019). Future Needs in Mast Cell Biology. Int. J. Mol. Sci..

[40] Li Z., Liu S., Xu J., Zhang X., Han D., Liu J., Xia M., Yi L., Shen Q., Xu S. (2018). Adult Connective Tissue-Resident Mast Cells Originate from Late Erythro-Myeloid Progenitors. Immunity.

[41] Gentek R., Ghigo C., Hoeffel G., Bulle M.J., Msallam R., Gautier G., Launay P., Chen J., Ginhoux F., Bajenoff M. (2018). Hemogenic Endothelial Fate Mapping Reveals Dual Developmental Origin of Mast Cells. Immunity.

[42] Nilsson G., Dahlin J.S. (2019). New insights into the origin of mast cells. Allergy.

[43] Mantovani A., Marchesi F., Malesci A., Laghi L., Allavena P. (2017). Tumour-associated macrophages as treatment targets in oncology. Nat. Rev. Clin. Oncol..

[44] Binnewies M., Mujal A.M., Pollack J.L., Combes A.J., Hardison E.A., Barry K.C., Tsui J., Ruhland M.K., Kersten K., Abushawish M.A. (2019). Unleashing Type-2 Dendritic Cells to Drive Protective Antitumor CD4(+) T Cell Immunity. Cell.

[45] Galli S.J., Borregaard N., Wynn T.A. (2011). Phenotypic and functional plasticity of cells of innate immunity: Macrophages, mast cells and neutrophils. Nat. Immunol..

[46] Nicolas-Avila J.A., Adrover J.M., Hidalgo A. (2017). Neutrophils in Homeostasis, Immunity, and Cancer. Immunity.

[47] Kirshenbaum A.S., Yin Y., Sundstrom B., Bandara G., Metcalfe D.D. (2019). Description and Characterization of a Novel Human Mast Cell Line for Scientific Study. Int. J. Mol. Sci..

[48] Shefler I., Salamon P., Mekori Y.A. (2019). MicroRNA Involvement in Allergic and Non-Allergic Mast Cell Activation. Int. J. Mol. Sci..

[49] Theoharides T.C., Tsilioni I., Conti P. (2019). Mast Cells May Regulate the Anti-Inflammatory Activity of IL-37. Int. J. Mol. Sci..

[50] Kwon S.Y., Kim J.H. (2019). Role of Leukotriene B4 Receptor-2 in Mast Cells in Allergic Airway Inflammation. Int. J. Mol. Sci..

[51] Patella V., Marino I., Lamparter B., Arbustini E., Adt M., Marone G. (1995). Human heart mast cells. Isolation, purification, ultrastructure, and immunologic characterization. J. Immunol..

[52] Patella V., Marino I., Arbustini E., Lamparter-Schummert B., Verga L., Adt M., Marone G. (1998). Stem cell factor in mast cells and increased mast cell density in idiopathic and ischemic cardiomyopathy. Circulation.

[53] Syvaranta S., Helske S., Lappalainen J., Kupari M., Kovanen P.T. (2012). Lymphangiogenesis in aortic valve stenosis—Novel regulatory roles for valvular myofibroblasts and mast cells. Atherosclerosis.

[54] Parente R., Pucino V., Magliacane D., Petraroli A., Loffredo S., Marone G., Triggiani M. (2017). Evaluation of vaccination safety in children with mastocytosis. Pediatr. Allergy Immunol..

[55] Volertas S., Schuler C.F., Akin C. (2018). New Insights into Clonal Mast Cell Disorders Including Mastocytosis. Immunol. Allergy Clin. North Am..

[56] Weiler C.R., Austen K.F., Akin C., Barkoff M.S., Bernstein J.A., Bonadonna P., Butterfield J.H., Carter M., Fox C.C., Maitland A. (2019). AAAAI Mast Cell Disorders Committee Work Group Report: Mast cell activation syndrome (MCAS) diagnosis and management. J. Allergy Clin. Immunol..

[57] Valent P., Akin C., Gleixner K.V., Sperr W.R., Reiter A., Arock M., Triggiani M. (2019). Multidisciplinary Challenges in Mastocytosis and How to Address with Personalized Medicine Approaches. Int. J. Mol. Sci..

[58] Williams M., Lidke D.S., Hartmann K., George T.I. (2019). PD-L1 Expression in Mastocytosis. Int. J. Mol. Sci..

[59] Varricchi G., Galdiero M.R., Mercurio V., Bonaduce D., Marone G., Tocchetti C.G. (2018). Pharmacovigilating cardiotoxicity of immune checkpoint inhibitors. Lancet Oncol..

[60] Varricchi G., Galdiero M.R., Tocchetti C.G. (2017). Cardiac Toxicity of Immune Checkpoint Inhibitors: Cardio-Oncology Meets Immunology. Circulation.

[61] Lavin Y., Kobayashi S., Leader A., Amir E.D., Elefant N., Bigenwald C., Remark R., Sweeney R., Becker C.D., Levine J.H. (2017). Innate Immune Landscape in Early Lung Adenocarcinoma by Paired Single-Cell Analyses. Cell.

[62] Nakae S., Suto H., Iikura M., Kakurai M., Sedgwick J.D., Tsai M., Galli S.J. (2006). Mast cells enhance T cell activation: Importance of mast cell costimulatory molecules and secreted TNF. J. Immunol..

[63] Hatch E.W., Geeze M.B., Martin C., Salama M.E., Hartmann K., Eisenwort G., Blatt K., Valent P., Gotlib J., Lee J.H. (2018). Variability of PD-L1 expression in mastocytosis. Blood Adv..

[64] Varricchi G., Pecoraro A., Marone G., Criscuolo G., Spadaro G., Genovese A. (2018). Thymic Stromal Lymphopoietin Isoforms, Inflammatory Disorders, and Cancer. Front. Immunol..

[65] Chen C.H., Ferreira J.C., Gross E.R., Mochly-Rosen D. (2014). Targeting aldehyde dehydrogenase 2: New therapeutic opportunities. Physiol. Rev..

[66] Goedde H.W., Agarwal D.P., Fritze G., Meier-Tackmann D., Singh S., Beckmann G., Bhatia K., Chen L.Z., Fang B., Lisker R. (1992). Distribution of ADH2 and ALDH2 genotypes in different populations. Hum. Genet..

[67] Harada S., Agarwal D.P., Goedde H.W. (1981). Aldehyde dehydrogenase deficiency as cause of facial flushing reaction to alcohol in Japanese. Lancet.

[68] Kim D.K., Cho Y.E., Song B.J., Kawamoto T., Metcalfe D.D., Olivera A. (2019). Aldh2 Attenuates Stem Cell Factor/Kit-Dependent Signaling and Activation in Mast Cells. Int. J. Mol. Sci..

[69] Galdiero M.R., Varricchi G., Seaf M., Marone G., Levi-Schaffer F. (2017). Bidirectional Mast Cell-Eosinophil Interactions in Inflammatory Disorders and Cancer. Front. Med..

[70] Visciano C., Liotti F., Prevete N., Cali G., Franco R., Collina F., de Paulis A., Marone G., Santoro M., Melillo R.M. (2015). Mast cells induce epithelial-to-mesenchymal transition and stem cell features in human thyroid cancer cells through an IL-8-Akt-Slug pathway. Oncogene.

[71] Yu Y., Blokhuis B.R., Garssen J., Redegeld F.A. (2019). A Transcriptomic Insight into the Impact of Colon Cancer Cells on Mast Cells. Int. J. Mol. Sci..

[72] Sammarco G., Gadaleta C.D., Zuccala V., Albayrak E., Patruno R., Milella P., Sacco R., Ammendola M., Ranieri G. (2018). Tumor-Associated Macrophages and Mast Cells Positive to Tryptase Are Correlated with Angiogenesis in Surgically-Treated Gastric Cancer Patients. Int. J. Mol. Sci..

[73] Ammendola M., Sacco R., Vescio G., Zuccala V., Luposella M., Patruno R., Zizzo N., Gadaleta C., Marech I., Ruggieri R. (2017). Tryptase mast cell density, protease-activated receptor-2 microvascular density, and classical microvascular density evaluation in gastric cancer patients undergoing surgery: Possible translational relevance. Ther. Adv. Gastroenterol..

[74] Ferrari S.M., Fallahi P., Galdiero M.R., Ruffilli I., Elia G., Ragusa F., Paparo S.R., Patrizio A., Mazzi V., Varricchi G. (2019). Immune and Inflammatory Cells in Thyroid Cancer Microenvironment. Int. J. Mol. Sci..

[75] Ribatti D., Tamma R., Vacca A. (2019). Mast Cells and Angiogenesis in Human Plasma Cell Malignancies. Int. J. Mol. Sci..

[76] Gorzalczany Y., Sagi-Eisenberg R. (2019). Role of Mast Cell-Derived Adenosine in Cancer. Int. J. Mol. Sci..

[77] Babina M., Wang Z., Franke K., Guhl S., Artuc M., Zuberbier T. (2019). Yin-Yang of IL-33 in Human Skin Mast Cells: Reduced Degranulation, but Augmented Histamine Synthesis through p38 Activation. J. Investig. Dermatol..

[78] Ravindran A., Ronnberg E., Dahlin J.S., Mazzurana L., Safholm J., Orre A.C., Al-Ameri M., Peachell P., Adner M., Dahlen S.E. (2018). An Optimized Protocol for the Isolation and Functional Analysis of Human Lung Mast Cells. Front. Immunol..

[79] Saadalla A.M., Osman A., Gurish M.F., Dennis K.L., Blatner N.R., Pezeshki A., McNagny K.M., Cheroutre H., Gounari F., Khazaie K. (2018). Mast cells promote small bowel cancer in a tumor stage-specific and cytokine-dependent manner. Proc. Natl. Acad. Sci. USA.

[80] Paupert J., Espinosa E., Cenac N., Robert V., Laharrague P., Evrard S.M., Casteilla L., Lorsignol A., Cousin B. (2018). Rapid and Efficient Production of Human Functional Mast Cells through a Three-Dimensional Culture of Adipose Tissue-Derived Stromal Vascular Cells. J. Immunol..

[81] Gordon J.R., Galli S.J. (1990). Mast cells as a source of both preformed and immunologically inducible TNF-alpha/cachectin. Nature.

[82] Zhang B., Alysandratos K.D., Angelidou A., Asadi S., Sismanopoulos N., Delivanis D.A., Weng Z., Miniati A., Vasiadi M., Katsarou-Katsari A. (2011). Human mast cell degranulation and preformed TNF secretion require mitochondrial translocation to exocytosis sites: Relevance to atopic dermatitis. J. Allergy Clin. Immunol..

[83] Benyon R.C., Bissonnette E.Y., Befus A.D. (1991). Tumor necrosis factor-alpha dependent cytotoxicity of human skin mast cells is enhanced by anti-IgE antibodies. J. Immunol..

[84] Jawdat D.M., Rowden G., Marshall J.S. (2006). Mast cells have a pivotal role in TNF-independent lymph node hypertrophy and the mobilization of Langerhans cells in response to bacterial peptidoglycan. J. Immunol..

[85] Okayama Y., Hagaman D.D., Metcalfe D.D. (2001). A comparison of mediators released or generated by IFN-gamma-treated human mast cells following aggregation of Fc gamma RI or Fc epsilon RI. J. Immunol..

[86] Nakamura Y., Franchi L., Kambe N., Meng G., Strober W., Nunez G. (2012). Critical role for mast cells in interleukin-1beta-driven skin inflammation associated with an activating mutation in the nlrp3 protein. Immunity.

[87] Kanaoka Y., Austen K.F. (2019). Roles of cysteinyl leukotrienes and their receptors in immune cell-related functions. Adv. Immunol..

[88] Vigorito C., Giordano A., Cirillo R., Genovese A., Rengo F., Marone G. (1997). Metabolic and hemodynamic effects of peptide leukotriene C4 and D4 in man. Int. J. Clin. Lab. Res..

[89] Wakahara S., Fujii Y., Nakao T., Tsuritani K., Hara T., Saito H., Ra C. (2001). Gene expression profiles for Fc epsilon RI, cytokines and chemokines upon Fc epsilon RI activation in human cultured mast cells derived from peripheral blood. Cytokine.

[90] Al-Afif A., Alyazidi R., Oldford S.A., Huang Y.Y., King C.A., Marr N., Haidl I.D., Anderson R., Marshall J.S. (2015). Respiratory syncytial virus infection of primary human mast cells induces the selective production of type I interferons, CXCL10, and CCL4. J. Allergy Clin. Immunol..

[91] Burke S.M., Issekutz T.B., Mohan K., Lee P.W., Shmulevitz M., Marshall J.S. (2008). Human mast cell activation with virus-associated stimuli leads to the selective chemotaxis of natural killer cells by a CXCL8-dependent mechanism. Blood.

[92] Joulia R., L’Faqihi F.E., Valitutti S., Espinosa E. (2017). IL-33 fine tunes mast cell degranulation and chemokine production at the single-cell level. J. Allergy Clin. Immunol..

[93] Chang Y.L., Wang Z., Igawa S., Choi J.E., Werbel T., Di Nardo A. (2019). Lipocalin 2: A New Antimicrobial in Mast Cells. Int. J. Mol. Sci..

[94] Frossi B., De Carli M., Calabro A. (2019). Coeliac Disease and Mast Cells. Int. J. Mol. Sci..

[95] Maki M., Mearin M.L., Polanco I., Schmitz J., Troncone R. (2018). Chapter 5.1.1. Coeliac Disease. J. Pediatr. Gastroenterol. Nutr..

[96] Korponay-Szabo I.R., Troncone R., Discepolo V. (2015). Adaptive diagnosis of coeliac disease. Best Pract. Res. Clin. Gastroenterol..

[97] Smolen J.S., Aletaha D., McInnes I.B. (2016). Rheumatoid arthritis. Lancet.

[98] de Paulis A., Marino I., Ciccarelli A., de Crescenzo G., Concardi M., Verga L., Arbustini E., Marone G. (1996). Human synovial mast cells. I. Ultrastructural in situ and in vitro immunologic characterization. Arthritis Rheum..

[99] Gotis-Graham I., McNeil H.P. (1997). Mast cell responses in rheumatoid synovium. Association of the MCTC subset with matrix turnover and clinical progression. Arthritis Rheum..

[100] Gotis-Graham I., Smith M.D., Parker A., McNeil H.P. (1998). Synovial mast cell responses during clinical improvement in early rheumatoid arthritis. Ann. Rheum. Dis..

[101] Lee D.M., Friend D.S., Gurish M.F., Benoist C., Mathis D., Brenner M.B. (2002). Mast cells: A cellular link between autoantibodies and inflammatory arthritis. Science.

[102] Pitman N., Asquith D.L., Murphy G., Liew F.Y., McInnes I.B. (2011). Collagen-induced arthritis is not impaired in mast cell-deficient mice. Ann. Rheum. Dis..

[103] Zhou J.S., Xing W., Friend D.S., Austen K.F., Katz H.R. (2007). Mast cell deficiency in Kit (W-sh) mice does not impair antibody-mediated arthritis. J. Exp. Med..

[104] Undem B.J., Taylor-Clark T. (2014). Mechanisms underlying the neuronal-based symptoms of allergy. J. Allergy Clin. Immunol..

[105] Veiga-Fernandes H., Artis D. (2018). Neuronal-immune system cross-talk in homeostasis. Science.

[106] Forsythe P., Bienenstock J. (2012). The mast cell-nerve functional unit: A key component of physiologic and pathophysiologic responses. Chem. Immunol. Allergy.

[107] Patel A.B., Tsilioni I., Leeman S.E., Theoharides T.C. (2016). Neurotensin stimulates sortilin and mTOR in human microglia inhibitable by methoxyluteolin, a potential therapeutic target for autism. Proc. Natl. Acad. Sci. USA.

[108] Zhang X., Wang Y., Dong H., Xu Y., Zhang S. (2016). Induction of Microglial Activation by Mediators Released from Mast Cells. Cell. Physiol. Biochem..

[109] Theoharides T.C., Stewart J.M., Panagiotidou S., Melamed I. (2016). Mast cells, brain inflammation and autism. Eur. J. Pharmacol..

[110] Skaper S.D., Facci L., Giusti P. (2014). Neuroinflammation, microglia and mast cells in the pathophysiology of neurocognitive disorders: A review. CNS Neurol. Disord. Drug Targets.

[111] Girolamo F., Coppola C., Ribatti D. (2017). Immunoregulatory effect of mast cells influenced by microbes in neurodegenerative diseases. Brain Behav. Immun..

[112] Theoharides T.C., Kavalioti M., Tsilioni I. (2019). Mast Cells, Stress, Fear and Autism Spectrum Disorder. Int. J. Mol. Sci..

[113] Boo B., Kamath R., Arriaga-Gomez E., Landry J., Emanuel E., Joo S., Saldias Montivero M., Martinov T., Fife B.T., Chatterjea D. (2019). Tetrahydrocannabinol Reduces Hapten-Driven Mast Cell Accumulation and Persistent Tactile Sensitivity in Mouse Model of Allergen-Provoked Localized Vulvodynia. Int. J. Mol. Sci..

[114] Marichal T., Starkl P., Reber L.L., Kalesnikoff J., Oettgen H.C., Tsai M., Metz M., Galli S.J. (2013). A beneficial role for immunoglobulin E in host defense against honeybee venom. Immunity.

[115] Starkl P., Marichal T., Gaudenzio N., Reber L.L., Sibilano R., Tsai M., Galli S.J. (2016). IgE antibodies, FcepsilonRIalpha, and IgE-mediated local anaphylaxis can limit snake venom toxicity. J. Allergy Clin. Immunol..

[116] Wernersson S., Pejler G. (2014). Mast cell secretory granules: Armed for battle. Nat. Rev. Immunol..

[117] Dwyer D.F., Barrett N.A., Austen K.F. (2016). Expression profiling of constitutive mast cells reveals a unique identity within the immune system. Nat. Immunol..

[118] Grootens J., Ungerstedt J.S., Nilsson G., Dahlin J.S. (2018). Deciphering the differentiation trajectory from hematopoietic stem cells to mast cells. Blood Adv..

[119] Aran D., Looney A.P., Liu L., Wu E., Fong V., Hsu A., Chak S., Naikawadi R.P., Wolters P.J., Abate A.R. (2019). Reference-based analysis of lung single-cell sequencing reveals a transitional profibrotic macrophage. Nat. Immunol..

[120] Wen T., Aronow B.J., Rochman Y., Rochman M., Kc K., Dexheimer P.J., Putnam P., Mukkada V., Foote H., Rehn K. (2019). Single-cell RNA sequencing identifies inflammatory tissue T cells in eosinophilic esophagitis. J. Clin. Investig..

[121] Thommen D.S., Koelzer V.H., Herzig P., Roller A., Trefny M., Dimeloe S., Kiialainen A., Hanhart J., Schill C., Hess C. (2018). A transcriptionally and functionally distinct PD-1(+) CD8(+) T cell pool with predictive potential in non-small-cell lung cancer treated with PD-1 blockade. Nat. Med..

[122] Bradding P., Arthur G. (2016). Mast cells in asthma—State of the art. Clin. Exp. Allergy.

[123] Castells M., Butterfield J. (2019). Mast Cell Activation Syndrome and Mastocytosis: Initial Treatment Options and Long-Term Management. J. Allergy Clin. Immunol. Pract..

[124] Brown M.A., Weinberg R.B. (2018). Mast Cells and Innate Lymphoid Cells: Underappreciated Players in CNS Autoimmune Demyelinating Disease. Front. Immunol..

[125] Yu X., Kasprick A., Hartmann K., Petersen F. (2018). The Role of Mast Cells in Autoimmune Bullous Dermatoses. Front. Immunol..

[126] Meixiong J., Anderson M., Limjunyawong N., Sabbagh M.F., Hu E., Mack M.R., Oetjen L.K., Wang F., Kim B.S., Dong X. (2019). Activation of Mast-Cell-Expressed Mas-Related G-Protein-Coupled Receptors Drives Non-histaminergic Itch. Immunity.

[127] Taracanova A., Tsilioni I., Conti P., Norwitz E.R., Leeman S.E., Theoharides T.C. (2018). Substance P and IL-33 administered together stimulate a marked secretion of IL-1beta from human mast cells, inhibited by methoxyluteolin. Proc. Natl. Acad. Sci. USA.

